# Human Milk Omega-3 Fatty Acid Composition Is Associated with Infant Temperament

**DOI:** 10.3390/nu11122964

**Published:** 2019-12-04

**Authors:** Jennifer Hahn-Holbrook, Adi Fish, Laura M. Glynn

**Affiliations:** 1 Department of Psychology, University of California, Merced, 5200 North Lake Rd, Merced, CA 95343, Canada; afish@ucmerced.edu; 2 Department of Psychology, Chapman University, Orange, CA 92866, USA; lglynn@chapman.edu

**Keywords:** breastfeeding, breast milk, temperament, fatty acids, LC-PUFA, omega-3, omega-6, DHA, AA, children, early life nutrition

## Abstract

There is growing evidence that omega-3 (n-3) polyunsaturated fatty-acids (PUFAs) are important for the brain development in childhood and are necessary for an optimal health in adults. However, there have been no studies examining how the n-3 PUFA composition of human milk influences infant behavior or temperament. To fill this knowledge gap, 52 breastfeeding mothers provided milk samples at 3 months postpartum and completed the Infant Behavior Questionnaire (IBQ-R), a widely used parent-report measure of infant temperament. Milk was assessed for n-3 PUFAs and omega-6 (n-6) PUFAs using gas-liquid chromatography. The total fat and the ratio of n-6/n-3 fatty acids in milk were also examined. Linear regression models revealed that infants whose mothers’ milk was richer in n-3 PUFAs had lower scores on the negative affectivity domain of the IBQ-R, a component of temperament associated with a risk for internalizing disorders later in life. These associations remained statistically significant after considering covariates, including maternal age, marital status, and infant birth weight. The n-6 PUFAs, n-6/n-3 ratio, and total fat of milk were not associated with infant temperament. These results suggest that mothers may have the ability to shape the behavior of their offspring by adjusting the n-3 PUFA composition of their milk.

## 1. Introduction

Early life nutrition plays a foundational role in brain development [1,2,3]. In recent decades, research has shown that exposure to human milk and the variation in its composition contribute meaningfully to children’s behavior, cognition, and disease risk [4,5,6]. The American Academy of Pediatrics recommends that human milk be the sole source of infant nutrition for the first 6 months of life [7], a sensitive period characterized by rapid brain development [2,3]. One key nutritional factor that is present in human milk and that is necessary for an optimal brain development during this period are omega-3 (n-3) polyunsaturated fatty-acids (PUFAs) [8,9]. 

Human milk contains relatively high levels of n-3 PUFAs, which are essential to visual, motor, and cognitive development [8,9]. Among the 11 n-3 PUFAs, the three most important and prevalent in human milk are alpha-linolenic acid (ALA), eicosapentaenoic acid (EPA), and docosahexaenoic acid (DHA) [5,10]. ALA is the most common n-3 PUFA in human milk [10] (and the adult diet) and can be converted into EPA and DHA [11]. DHA is the most abundant n-3 PUFA in the central nervous system in mammals and forms the structural matrix of grey matter and retinal membranes [9,12]. EPA is used to produce eicosanoids, signaling molecules that play numerous roles, including reducing inflammation in the body and the brain [13,14]. The U.S. Department of Health and Human Services 2015−2020 Dietary Guidelines for Americans recommend that all adults, particularly pregnant and breastfeeding women, consume 8 ounces of seafood per week, providing approximately 250 mg of EPA and DHA per day [15]. Breast-fed infants have significantly higher levels of n-3 PUFAs in their plasma lipids at three months than do infants given formula lacking n-3 fortification [16]. An autopsy study showed that the rate of accumulation of DHA is approximately 5.0 mg a day in the brains of breast-fed infants versus 2.3 mg a day in infants fed non-DHA fortified formula [17].

Numerous studies have shown that deficiencies in n-3 PUFAs are related to mood and anxiety disorders [18,19]. In adults, n-3 supplementation has benefits for the prevention and treatment of major depression [20], bipolar disorder [21], and anxiety disorders [22]. Much less is known about how early variations in the exposure to n-3 PUFAs impact the mood and behavior of children. However, animal models suggest that early n-3 exposure can have a lasting impact on offspring temperament and behavioral phenotypes. For example, feeding pregnant rats diets deficient in n-3 PUFAs increases anxiety-like behavior in rat pups, and upregulates anxiogenic-related glucocorticoid receptors in the frontal cortex, hypothalamus, and hippocampus [23]. These pre-clinical studies are consistent with correlational studies in human children. For example, in a diverse study of 255 women, eating a diet higher in n-3 relative to n-6 fatty acids during pregnancy buffered Black infants against the detrimental effects of maternal stress on infant regulatory capacities [24]. Plasma DHA levels have also been found to negatively correlate with depressive symptoms in children and adolescents with bipolar disorder [25].

No studies, however, could be found examining whether the n-3 PUFA composition of milk influences the infant mood or anxiety in humans, although several studies have linked cortisol levels in milk to infant temperament [26,27,28]. For example, one study showed that higher levels of cortisol in milk predicted an enhanced performance on the autonomic stability cluster on the Neonatal Behavioral Assessment Scale in neonates [27]. Studies have also found that cortisol levels in mothers’ milk are positively correlated with a negative affectivity and fear reactivity in humans [26,28]. Most of what we know about lactational programing comes from animal models, and findings are generally consistent with the notion that milk is an important early moderator of the infant phenotype [29,30,31]. For example, in a study of Rhesus Macaques, heavier mothers with more reproductive experience were able to supply more calories to their infant through milk [32]. Moreover, infants whose mothers supplied more calories through milk in the early postnatal period showed higher activity levels and a greater confidence in a stressful setting later in infancy [32]. In sum, research has neglected the question of whether the fatty acid composition of milk influences infant temperament in humans.

To fill this gap, the current study tests whether the n-3 PUFA composition of mothers’ milk is associated with the temperament of their infants. To address this question, milk samples were collected from 52 mothers of 3-month old infants and assessed for n-3 PUFA levels using gas-liquid chromatography. Mothers also completed the Rothbart Revised Infant Behavior Questionnaire (IBQ-R) [33], a widely-used parental-report instrument that assesses three broad dimensions of infant temperament (negative affectivity, orienting/regulation, and surgency/extraversion). Given that n-3 PUFAs and higher n-3/n-6 ratios have been found to be protective against mood disorders and anxiety symptoms [20], we predicted that n-3 PUFAs and the n-3/n-6 ratio in milk would be inversely correlated with the negative affectivity dimension of the IBQ-R. n-3 PUFAs have also been linked to enhanced executive functioning in children [9]. Therefore, we predicted that milk n-3 PUFAs levels would be positively correlated with scores on the orienting/regulation temperament dimension. We had no predictions regarding the surgency/extraversion temperament dimension. Exploratory analyses are also presented; they test whether the levels of n-6 PUFAs, total PUFAs or total milk fat concentrations are associated with infant temperament.

## 2. Methods

### 2.1. Participants

Fifty-two breastfeeding mothers and their three-month-old infants were enrolled in a large longitudinal study of early development from a medical center in Southern California. To be eligible to participate in the study, participants had to be over the age of 18, English-speaking, with a singleton intrauterine pregnancy. Women were ineligible for this study if they used alcohol, tobacco, illicit drugs, had cervical or uterine abnormalities, used medications that impacted the endocrine function, had a diagnosis of a disease influencing the neuroendocrine function, or had an infant admittance to the Neonatal Intensive Care Unit at birth because of compromised health (e.g., intrauterine growth restriction and respiratory distress syndrome).

After mothers gave informed consent to participate in the study, they were asked to come into the laboratory at 3 months postpartum (M = 3.01 months, SD = 0.25) to provide milk samples and fill out a survey regarding their infants’ temperament. This study was approved by the Institutional Review Board at the University of California, Irvine (ethics approval code HS# 2002−2441, first approved: January 31, 2003; renewed most recently: 25 February 2019).

### 2.2. Determination of Milk Fatty Acid Levels

Mothers were asked to empty the contents of one breast with an electric breast pump into a sterile plastic container (Medela, Inc., McHenry, IL, USA). In an effort to ensure that the mother’s milk donation for the study would not adversely impact infant nutrition, providing milk samples was made an optional part of this larger longitudinal study, and mothers were only asked to empty the contents of one breast, leaving milk in the other breast for the infant. Before the collection, the mothers were asked to clean the breast and nipple area with an antibacterial wipe and wait until the area dried (to leave open the possibility to examine the microbial composition of the milk for later research). The milk samples were then immediately aliquoted into polypropylene tubes and stored at 70 °C until they were assessed for fatty acids. The time of day of the milk collection was recorded and modestly correlated with the n-3 fatty acid composition of the milk (Pearson’s *r* = −0.344, *p* = 0.015). The time of day of the sample collection was not related to any of the other fatty acids’ composite variables (Pearson’s *r* ranged from −0.175 to −0.008; *ps* > 0.153).

The fatty acid composition of the breast milk was analyzed by gas-liquid chromatography in prepared fatty acid methyl esters. The total lipids were extracted from the milk by a method described previously [34], using tridecanoin as the internal standard [35]. The thawed milk samples were shaken vigorously and saponified in 6% ethanolic KOH for 1 h at 37 °C. The mixture was extracted twice with hexane (20 min on rotator), and spun to separate the phases. The hexane layers were then combined and evaporated under a gentle stream of nitrogen to reduce lipid peroxidation. The fatty acid methyl esters were prepared by heating the reconstituted samples in 12% boron trifluoride-methanol for 10 min at 100 °C in tightly sealed tubes. The derivatized samples were separated with a Hewlett-Packard gas chromatograph equipped with an Omegawax 250 capillary column and isothermal oven. 

The total of the n-3 PUFAs was computed by summing the total fats from ALA, EPA, DHA, and a rare n-3 PUFA detected in milk, Eicosatrienoic acid (ETE). The total of the n-6 fatty acids was computed by summing the total fats from Linolelaidic acid, Arachidic acid, Linoleic acid, Linolenic acid, Dihomo-gamma-linolenic acid (DGLA), Arachidonic acid (AA), and Eicosadienoic Acid. The n-6/n-3 PUFA ratio was computed by dividing the total fats from n-6 PUFAs by the total fats from n-3 PUFAs. Higher ratios represent milk that has more n-6 fatty acids relative to n-3s. The total PUFA concentration in milk was determined by summing the levels of n-3 and n-6 PUFAs.

### 2.3. Infant Temperament

To assess the infant temperament, mothers were asked to fill out the Rothbart Revised Infant Behavior Questionnaire (IBQ-R) during their laboratory visit [33]. The IBQ-R includes 191 specific questions addressing concrete behaviors such as, “During a peek-a-boo game, how often did the baby smile?” and “How often during the last week did the baby startle to a sudden or loud noise?” To prevent errors in recall, the scale only asks mothers about recently occurring events, using a 7-point Likert scale (1-never to 7-always). The IBQ-R measures three broad dimensions of temperament: negative affectivity, surgency/extraversion, and orienting/regulation. The negative affectivity dimension is created by averaging the scores across four subscales assessing infant sadness, fear, falling reactivity, and distress to limitations. The orienting/regulation dimension is created by averaging across four subscales of mothers’ ratings of infants’ cuddliness/affiliation, low intensity pleasure, duration of orienting, and soothability. The surgency/extraversion dimension is a composite averaged from six subscales assessing infant approach, vocal reactivity, high intensity pleasure, smiling and laughter, activity level, and perceptual sensitivity. This widely used parental-report instrument has been shown to be reliable across parental reports [33] and to correlate well with behavioral observations of infants [36]. The IBQ-R has been validated for use for infants aged 3 to 12 months, scores are relatively stable over the first year of life [36], and the scale takes approximately 30 min to complete [33]. As part of the larger longitudinal study, mothers also completed the IBQ-R at 6 months. See Table S1 in the Supplemental Materials for exploratory analyses testing the association between the milk fatty acid composition at 3 months and the infant temperament at 6 months. 

### 2.4. Demographic and Health Information

Various demographic and health measures were tested as potential covariates. Maternal reports of race/ethnicity, age, education level, income, parity, marital status, and breastfeeding exclusivity were collected during a structured interview. A medical record review was conducted to assess the birth outcomes, including the infant sex, gestational age at delivery, and birth weight and length. The early pregnancy BMI (at 15 weeks) and weight gain during pregnancy (weight in pounds at 37 weeks-15 weeks) were collected during laboratory visits as part of the larger longitudinal study. At 3 months, the infant weight was assessed using a digital scale (Midmark, Versailles, OH, USA), and the height was determined while the child lay in a supine position. The child BMI percentiles (BMIP) at birth and 3 months were calculated using an SPSS macro that fits the child’s height and weight to standard WHO growth curves and generates a child’s BMI z-score standardized for age and sex [37,38]. For ease of interpretation, these z-scores were converted to percentiles. 

### 2.5. Statistical Analysis Strategy

First, preliminary analyses were performed to check that the variables were normally distributed. Outliers that were greater than 3 standard deviations above or below the mean were winsorized to bring them to within 2 standard deviations before the analysis. We planned to log-transform any skewed variables; however, after winsorization, all variables were normally distributed. Second, we sought to identify potential confounds by testing whether demographic or health characteristics were associated with fatty acid concentrations in milk. Linear regression models were run with various demographic (age, income, education, marital status, race/ethnicity and infant sex) and health (maternal BMI, weight gain in pregnancy, gestational age at birth, exclusive breastfeeding status, and infant BMIP at birth and at 3 months) covariates entered simultaneously to predict n-3, n-6, n-6/n-3 ratios, and the total milk fat. Demographic or health variables that were associated with any one of the milk fatty acid composite variables with a *p*-value < 0.10 were included as covariates in the subsequent analysis. For the primary analysis, a multivariate linear regression was used to test whether the n-3, n-6, n-6/n-3 ratios, total PUFAs, or total milk fat predicted the negative affectivity or orienting/regulation dimension of the IBQ-R, adjusting for potential confounds. We also ran an exploratory analysis to see whether the fatty acid composition of milk was associated with the surgency/extraversion factor. If there was a significant association, we then used a follow-up linear regression analysis to test: (i) which specific sub-scales that made up the IBQ-R temperament dimension were significantly related to the fatty acid composite, and (ii) which specific fatty acid type (e.g., DHA vs. ALA) predicted the IBQ-R temperament dimension. Finally, we tested whether infant sex or exclusive breastfeeding moderated any observed significant association between the fatty acid levels and IBQ-R dimension. A moderation analysis was carried out by creating cross products between the potential moderator (infant sex, exclusive breastfeeding) and fatty acid levels, and the cross-products were then included in a linear regression model with the constituent variables and covariates. All analyses were performed in SPSS version 21.0. The findings were considered statistically significant if the *p*-values were under 0.05. The effect sizes (Standardized Betas or β) are also provided for all analyses, regardless of the significance level. 

## 3. Results

### 3.1. Preliminary and Descriptive Analyses

The demographic and health information of the samples is presented in Table 1. The fatty acids composition of the milk samples are presented in Table 2. Several milk samples were more than three standard deviations higher than the mean in terms of n-3 PUFA levels (*n* = 1), EPA levels (*n* = 2), ALA levels (*n* = 1), and ETE levels (*n* = 1) and were winsorized to within 2 standard deviations of the mean before the statistical analysis.

See Table 1 for the results of the linear regression analysis testing for associations between sociodemographic and health factors and the milk fatty acid composition. Married mothers had significantly higher n-6/n-3 ratios in their milk than mothers who were not married (*p* = 0.010). Older mothers tended to have higher levels of n-3 PUFAs in their milk (*p* = 0.060). Babies with a higher birth weight also tended to have higher n-6/n-3 ratios in their milk (*p* = 0.086). Infant sex, gestational age at delivery, and infant BMIP at birth or 3 months were not associated with the milk fatty acid composition. Likewise, mothers’ pre-pregnancy BMI, weight gain in pregnancy, income, education, exclusive breastfeeding status, parity and race/ethnicity did not predict fatty acid levels in milk. Therefore, only the maternal age, marital status, and infant birth weight were included as covariates in the subsequent linear regression models. 

The average infant score on the surgency/extraversion factor was 3.92 (SD = 0.88), the negative affectivity factor was 2.99 (SD = 0.56), and the orienting regulation factor was 5.02 (SD = 0.62). These mean scores are similar to those reported in the original validation study that had a larger sample size [33].

### 3.2. Primary Analysis

**Figure 1:**
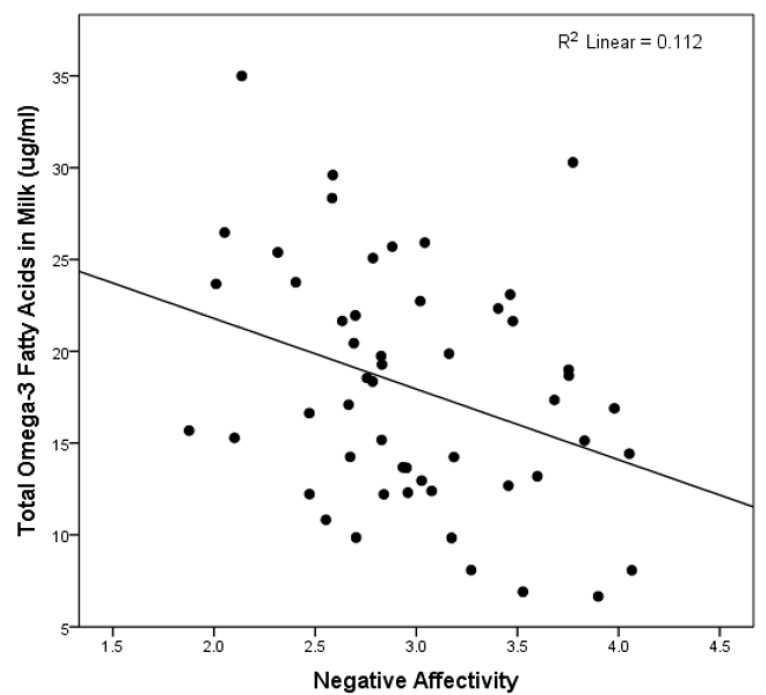
The scatterplot of the total omega-3 fatty acids in the milk and the infant negative affectivity.

See Table 3 for the results of the multivariate linear regression analyses testing the association between the fatty acid composite variables and the three primary temperament dimensions of the IBQ-R. These analyses revealed that only higher levels of n-3 PUFA levels predicted lower scores on the negative affectivity dimension (see Figure 1). This association remained statistically significant after adjustment for the total milk fat content (β = −0.443, *p* = 0.023) and when all covariates were removed from the model (β = −0.335, *p* = 0.015). In addition, controlling for the time of day of the milk collection did not change the significant association between n-3 fatty acids and negative affectivity.

Because the milk n-3 PUFA levels predicted a negative affectivity, we performed a follow-up analysis to test how the n-3 PUFAs predicted each of the four subscales that comprised this dimension of the IBQ-R. Higher n-3 PUFAs were associated with less sadness (β = −0.349, *p* = 0.021) and less distress to limitations (β = −365, *p* = 0.017). Milk n-3s were not significantly related to infant fear (β = −0.187, *p* = 0.219) or falling reactivity (β = −0.087, *p* = 0.584), although these effects were in the same direction as those observed for sadness and limitations to distress. 

In addition, given that n-3 PUFAs predicted negative affectivity, we tested whether specific subtypes of n-3 PUFAs (ALA, EPA, DHA, or ETE) were, independently, related to negative affectivity. Mothers with higher ALA in their milk reported that their infants had significantly less negative affectivity (β = −0.368, *p* = 0.015). Although they did not reach the criterion for statistical significance, the DHA, EPA, and ETE levels were all negatively associated with negative affectivity (DHA: β = −0.178, *p* = 0.229; EPA: β = −0.251, *p* = 0.095; ETE: β = −0.103, *p* = 0.472).

### 3.3. Moderation Analysis

Neither the infant sex, nor the breastfeeding exclusivity, significantly moderated the association between the milk n-3 PUFA levels and negative affectivity (sex: β = −0.170, *p* = 0.780; breastfeeding exclusivity: β = 0.975, *p* = 0.071). 

## 4. Discussion

The present study found evidence to support a link between the n-3 PUFA composition of human milk and infant temperament. Specifically, infants whose mothers had higher levels of n-3 PUFAs in their milk were reported to display lower levels of negative affectivity at 3 months than did infants whose mothers had lower levels of n-3 PUFAs in their milk. To the extent that negative affectivity in infancy is associated with a higher risk for internalizing disorders in later childhood [39,40], n-3 PUFAs in milk may represent a novel developmental origins pathway through which environmental exposures influence the risk for later mental health disorders. To our knowledge, this is the first study to report an association between the variation in the n-3 PUFAs levels in milk and the temperament in humans. One previous study did report that there was no difference in the temperaments of infants who were randomly assigned to receive formula supplemented with DHA (vs. non-DHA-supplemented formula) [41] (see [42] for similar null results with rhesus macaques). However, formula supplementation studies are not directly comparable to studies of human milk, since n-3 PUFAs are metabolized differently in human milk than they are in formula [43], and since the absorption of fatty acids from formula is highly dependent on the specific type of oil from which the n-3s were harvested [44]. Still, the results of this study should be considered preliminary until they can be replicated in larger studies. 

There are several potential physiological pathways through which n-3 PUFAs in milk could influence infant affect regulation and temperament. Most directly, n-3 PUFAs and their derivatives help to regulate neurotransmission, and animal studies suggest that deficiencies increase the serotonin 2 (5-HT_2_) and decrease dopamine 2 (D_2_) receptor density in the frontal cortex [45]. The upregulation of 5-HT_2_ and downregulation of D_2_ in the brain have been implicated in mood disorders [46]. Less directly, n-3 PUFAs might influence infant negative affectivity through inflammatory pathways [18,19,47]. n-3 PUFAs, and EPA especially, have anti-inflammatory properties and reduce the levels of pro-inflammatory cytokines [18,19,47]. In adults, we know from animal and human experimental studies that pro-inflammatory cytokines can readily cross the blood–brain barrier and induce sickness behavior characterized by depressive-like symptoms such as low mood, social withdrawal, and anxiety [48]. The extent to which n-3s PUFAs regulate inflammatory processes in early development is unknown, but future research could investigate if n-3 PUFAs’ levels in milk are related to inflammatory processes in infants and if, by doing so, they indirectly influence negative affectivity. 

It was notable that, of the three primary types of n-3 PUFAs in milk, only higher levels of ALA were significantly associated with negative affectivity. The DHA and EPA levels were also inversely correlated with negative affectivity, but not to a statistically significant degree. It would be premature for readers to conclude, however, that only ALA (and not DHA or EPA) in milk influences infant temperament given that all three of the primary n-3 PUFAs were negatively correlated with negative affectivity, with effect sizes in the small-to-medium range. Future studies that combine the methods used here with direct assessments of infants’ plasma levels of ALA, DHA, and EPA are necessary to address the question of whether it is n-3 PUFAs in general, or one subtype of n-3 PUFA in particular, that influences infant temperament. 

In their strongest form, these results could have several potential policy implications. First, the maternal diet can influence the levels of n-3 PUFAs in milk and, by doing so, may influence infant temperament. For example, breast milk EPA and DHA concentrations are closely linked to maternal dietary EPA and DHA intake [49], and randomized control trials have shown that mothers given n-3 PUFA supplements show corresponding increases in the n-3 PUFA composition of their milk [50,51]. Unfortunately, this study did not include information on maternal diets, and only two mothers in our study reported taking omega-3 PUFA supplements, so we were not able to test this hypothesis. Future studies could examine whether increasing the dietary intake of n-3 PUFAs in breastfeeding women would lead to a reduced negative affectivity in infants. Second, considering that negative affectivity in infancy predicts internalizing disorders (i.e., anxiety/depression) in later childhood [39,40], perhaps supplementing breastfeeding mothers with n-3 PUFAs could help ameliorate the risk for later mental health problems. While this possibility is only hypothetical, maternal diets during pregnancy and lactation are modifiable and present promising targets for the prevention of adverse developmental outcomes in children [52,53].

Although this study had several strengths, including the direct assessment of the fatty acid composition of milk and the carefully characterized cohort, these results should be considered in the context of several limitations. First, we relied on maternal reports of infant temperament, which may be biased. Despite this, the IBQ-R has in previous studies been found to correlate highly with more objective measures of infant temperament (e.g., behavioral observations) [36]. Moreover, the association between the reports of mothers and other caregivers is generally high [33]. Still, future research would benefit from including additional object measures of infant temperament and behavior. Second, this study was correlational and cannot rule out the possibility that a confounding factor may explain the observed association between the n-3 PUFA levels in milk and infant temperament. To address this possibility, we tested whether a number of demographic (e.g., income, education) and health (e.g., maternal BMI, weight gain in pregnancy) factors predicted the fatty acid composition of milk. Only the marital status, maternal age, and infant birth weight predicted the fatty acid composition of milk, and statistically adjusting for these covariates did not negate the association between n-3 PUFA levels and negative affectivity in this study. Regardless, there are numerous bio-active factors in milk, such as cortisol, which have been shown to correlate with total milk fat [54] and negative affectivity in infants [26]. However, given that we found no association between the total fat or n-6 fatty acid content of milk and infant temperament, we believe that the n-3 composition of milk and cortisol levels in milk likely represent distinct lactational programming pathways. Furthermore, the association between n-3 PUFA levels in milk and negative affectivity remains statistically significant after adjusting for the total fat content of the milk, suggesting that it is n-3 PUFAs specifically, and not richer milk generally, that shapes infant temperament. Finally, this study did not include information on the last time mothers had breastfed or on the total milk volume that mothers expressed. We were unable to identify any studies that tested how the time since the last feeding or the volume of milk production impacts the omega-3 fatty acid composition of milk. Future research is needed to determine whether these factors are associated with LC PUFAs and could therefore have influenced these results. 

Findings from this study add to the growing body of research demonstrating that the variation in the composition of mothers’ milk plays an important role in shaping the development of offspring. Specifically, we found that milk that was richer in n-3 PUFAs was associated with reduced sadness and distress in infants. These new data are consistent with previous research linking n-3 PUFAs to an improved mood and mental health in adults [18,19,20,21,22]. We hope that the exciting research taking place in the field of lactational programming will open up new possibilities for preventing mood disturbances. 

## Figures and Tables

**Table 1:** Sample characteristics and their association with the fatty acid composition of the milk.

	Mean/%	Standard Deviation	Range	Omega-3	Omega-6	Omega-6/3 Ratio	Total Fat
				Stand. β	Stand. β	Stand. β	Stand. β
**Maternal Characteristics**							
Maternal age	29.67	4.86	19.2–39.9	**0.407 ^t^**	0.285	−0.066	0.254
Education	2.70	1.051	0−4	0.113	−0.014	−0.167	−0.060
Household Income	69,489	33,980	25k–105k	0.008	−0.186	−0.286	−0.181
Pre-Pregnancy BMI	24.17	5.98	16.4–47.4	−0.102	−0.086	−0.045	−0.026
Pregnancy Weight Gain (lbs)	35.60	13.27	9.00–71.00	0.103	0.221	0.168	0.243
Parity (% Primiparous)	48.1%		1−4	0.085	−0.053	−0.123	−0.076
Exclusive Breastfeeding	63.27%			0.133	0.168	0.027	0.260
% Married	75%			−0.223	0.341	0.593 *	0.223
Race/Ethnicity							
% White	58.3%						
% Latina	18.8%			0.006	0.118	0.117	0.220
% Asian	10.4%			0.057	−0.006	−0.052	0.015
% Multi-Ethnic/Other	12.5%			−0.152	−0.011	0.170	−0.145
**Infant Characteristics**							
Birth Weight (grams)	3465.49	386.874	2470–4220	0.104	0.405	**0.449 ^t^**	0.215
Gestational Age at Birth (weeks)	39.6822	1.12	37.1–42.3	−0.093	−0.070	0.090	−0.147
BMIP at Birth	56.92%	0.30	0.34–98%	−0.114	−0.125	−0.175	−0.164
BMIP at 3 mos	46.25%	0.27	0.02–97%	0.164	0.101	−0.034	−0.013
% Female Infants	48%			0.117	0.181	0.035	0.120

Note: Race/ethnicity was dummy coded to create contrasts to compare Hispanic, Asian, and Multi-Ethnic/Other groups to whites. Married was coded as 1 = Married, 0 = Not married; Infant sex was coded as Female = 2, Male = 1. Bolded coefficients indicate those with *p*-values < 0.10 = t and *p*-values < 0.05 = *. BMIP = Body Mass Index Percentile.

**Table 2:** Milk fatty acid concentrations.

	IUPAC Name	Mean	Standard Deviation	Range
		(ug/mL)	(ug/mL)	(ug/mL)
**Omega-3 Fatty Acids**				
Linolenic acid (ALA)	(9Z,12Z,15Z)-octadeca-9,12,15-trienoic acid	13.80	6.15	4.26–42.25
Eicosatrienoic acid (ETE)	(11Z,14Z,17Z)-icosa-11,14,17-trienoic acid	0.74	1.02	0.02–7.00
Eicosapentaenoic acid (EPA)	(5Z,8Z,11Z,14Z,17Z)-icosa-5,8,11,14,17-pentaenoic acid	1.09	1.16	0.06–7.00
Docosahexaenoic acid (DHA)	(4Z,7Z,10Z,13Z,16Z,19Z)-docosa-4,7,10,13,16,19-hexaenoic acid	2.72	1.91	0.77–10.71
**Omega-6 Fatty Acids**				
Linolelaidic acid	(9E,12E)-octadeca-9,12-dienoic acid	1.16	0.30	1.00–3.08
Arachidic acid	(1^13^C)icosanoic acid	194.60	72.78	65.12–398.72
Linoleic acid	(9Z,12Z)-octadeca-9,12-dienoic acid	0.54	.06	0.51–0.89
Linolenic acid	(9Z,12Z,15Z)-octadeca-9,12,15-trienoic acid	2.26	1.71	0.14–8.79
Dihomo-gamma-linolenic acid (DGLA)	(8Z,11Z,14Z)-icosa-8,11,14-trienoic acid	5.41	2.21	1.41–10.55
Arachidonic acid (AA)	(5*Z*,8*Z*,11*Z*,14*Z*)-icosa-5,8,11,14-tetraenoic acid	1.17	0.94	0.12–4.92
Eicosadienoic Acid	(11E,14E)-icosa-11,14-dienoic acid	3.72	1.78	1.04–9.01
**Composite Fatty Acid Variables**				
Total Omega-3		18.36	7.98	6.66–55.64
Total Omega-6		208.85	77.88	70.11–433.86
Omega-6/Omega-3 Ratio		12.12	4.36	4.86–27.14
Total Milk PUFAs		227.21	83.57	83.58−489.50
Total Milk Fat		942.51	346.76	398–2301

**Table 3:** The association between the milk fatty acid composition and the infant temperament assessed with multivariate linear regression models.

	Negative Affectivity	Orienting/Regulation	Surgency/Extraversion
	Standardized β (*p*-Value)	Standardized β (*p*-Value)	Standardized β (*p*-Value)
Omega-3	−0.352 (0.020) *	−0.014 (0.927)	−0.108 (0.479)
Omega-6	−0.249 (0.106)	0.131 (0.394)	0.053 (0.727)
Omega-6/3 ratio	0.134 (0.387)	0.159 (0.297)	0.179 (0.236)
Total PUFAs	−0.266 (0.083)	0.127 (0.408)	0.042 (0.784)
Total Milk Fat	−0.124 (0.401)	−0.004 (0.981)	−0.039 (0.791)

Note: All coefficients are statistically adjusted for the maternal age, mother’s marital status, and infant birth weight. * *p* < 0.05.

## Supplementary Materials

The following are available online at https://www.mdpi.com/2072-6643/11/12/2964/s1, Table S1: Exploratory analysis of the association between the fatty-acid concentration of milk at 3-months and negative affectivity at 6-months (*n* = 44).
