# Supplementary material for: Human Milk Omega-3 Fatty Acid Composition Is Associated with Infant Temperament
Source: Nutrients. 2019 Dec 4;11(12):2964. doi: 10.3390/nu11122964 (PMC6949911; doi:10.3390/nu11122964)
Supplement: Supplementary file 1 [file nutrients-11-02964-s001.pdf]

Supplemental Materials

Table 1s. Exploratory analysis of the association between the fatty-acid concentration of milk at 3-months and negative affectivity at 6-months (N = 44)

	**Negative Affectivity**	**Negative Affectivity Subscales**			
		Sadness	Distress to Limitations	Fear	Falling Reactivity
	Standardized β (p-value)	*Standardized β (p-value)*	*Standardized β (p-value)*	*Standardized β (p-value)*	*Standardized β (p-value)*
Omega-3	-.216 (.187)	-.300 (.069)	-.113 (.499)	-.195 (.238)	-.032 (.848)
Omega-6	.056 (.735)	.025 (.881)	.053 (.753)	-.050 (.764)	.160 (.338)
n-6/3 ratio	.340 (.034)*	.400 (.013)*	.291 (.075)	.121 (.463)	.240 (.302)
Total PUFAs	.032 (.836)	-.002 (.989)	.038 (.891)	-.064 (.698)	.144 (.390)
Total Milk Fat	.090 (.568)	.054 (.735)	.127 (.424)	-.004 (.979)	.116 (.466)

*Note: All coefficients are statically adjusted for maternal age, mother's marital status, and infant birth weight. * $p < .05$.*
